# SAbDab in the age of biotherapeutics: updates including SAbDab-nano, the nanobody structure tracker

**DOI:** 10.1093/nar/gkab1050

**Published:** 2021-11-19

**Authors:** Constantin Schneider, Matthew I J Raybould, Charlotte M Deane

**Affiliations:** Department of Statistics, University of Oxford, Oxford, UK; Department of Statistics, University of Oxford, Oxford, UK; Department of Statistics, University of Oxford, Oxford, UK

## Abstract

In 2013, we released the Structural Antibody Database (SAbDab), a publicly available repository of experimentally determined antibody structures. In the interim, the rapid increase in the number of antibody structure depositions to the Protein Data Bank, driven primarily by increased interest in antibodies as biotherapeutics, has led us to implement several improvements to the original database infrastructure. These include the development of SAbDab-nano, a sub-database that tracks nanobodies (heavy chain-only antibodies) which have seen a particular growth in attention from both the academic and pharmaceutical research communities over the past few years. Both SAbDab and SAbDab-nano are updated weekly, comprehensively annotated with the latest features described here, and are freely accessible at opig.stats.ox.ac.uk/webapps/newsabdab/.

## INTRODUCTION

Antibodies are fundamental components of the immune system and represent the largest class of biotherapeutics; the 100th monoclonal antibody therapeutic has recently been approved by the FDA and the 20 most successful antibody therapeutics yielded a combined $110.6 billion in sales in 2019 (1). Their ability to bind to antigen targets of interest with high affinity and specificity make them promising candidates for the development of therapeutics against a variety of targets, including several cancer types and viruses such as SARS-CoV-2 (2,3).

**Figure 1.**
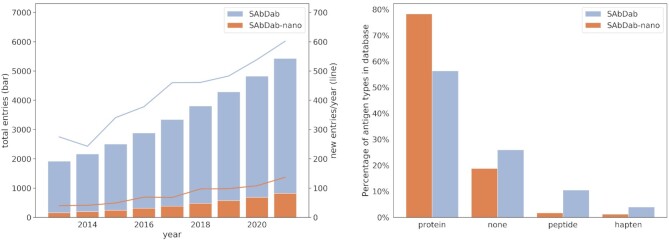
SAbDab-nano and SAbDab statistics. (Left) The number of entries in SAbDab and SAbDab-nano over time since the publication of the original SAbDab paper. The left y-axis and the bar plot depict the total number of entries in the respective databases at the end of the year, the right y-axis and the line plot depict the number of entries added to the databases in that year. Numbers for 2021 cover the time until 9 September 2021. Numbers for SAbDab include the content of SAbDab-nano, as SAbDab-nano is a subset of SAbDab. (Right) Antigen type composition of SAbDab and SAbDab-nano. Antigen types for which no structures exist in SAbDab-nano, but structures exist in SAbDab (carbohydrate, nucleic acid, other) are omitted. Protein here describes a polypeptide consisting of >30 amino acids, while peptide describes polypeptides consisting of less than 30 amino acids.

Due to the importance of an accurate understanding of the three-dimensional structure of antibodies for the study of their properties and the development of antibody therapeutics, we released the Structural Antibody Database (SAbDab) in 2013 (4), a comprehensive and continuously updated database of experimentally determined antibody structures. Since its publication, SAbDab has been used in numerous studies for the creation of tailored antibody datasets (5–7), and rapidly increased in size: from 1624 entries when it was published to 5426 at time of writing (see Figure 1).

In recent years, nanobodies, heavy chain-only antibodies which were first identified in camelids, have emerged as an important class of immune molecule. A large body of research has now been published on their properties and their potential as therapeutics (8,9), including for SARS-CoV-2 (10). As of August 2021, one nanobody therapeutic is approved and six more are in clinical trials (2). Meanwhile, the number of experimentally determined nanobody structures is also growing rapidly (see Figure 1).

While SAbDab has always contained nanobody structures, these recent trends have motivated the creation of SAbDab-nano, a sub-database of SAbDab which is the first nanobody-specific, continuously updated and annotated structure database. Here, we describe all our updates to SAbDab since its original publication, including SAbDab-nano.

## UPDATES TO DATA ANNOTATION

Using the annotation sources and protocols described in Dunbar *et al.* (4), each structure in SAbDab is annotated with the following: name, species, experimental method, resolution, amino acid sequence including the complementarity determining region (CDR) sequences as per the Chothia CDR definitions (11), the heavy and light chain subgroup, antigen type, and, where available, antigen binding affinity value (the latter is available for 746 entries in SAbDab, 87 of which are nanobodies). Over the last few years, we have created several auxiliary databases, including up-to-date collections of World Health Organisation-recognised therapeutic antibodies (Thera-SAbDab) (2) and coronavirus-binding antibodies (CoV-AbDab) (3). These databases contain antibody sequence information, linking to relevant entries in SAbDab where structures of the antibody in question (CoV-AbDab) or structures with at least 95% sequence identity (Thera-SAbDab) exist. Throughout SAbDab, we include the information contained in the auxiliary databases to annotate structures with a link to the relevant Thera-SAbDab and/or CoV-AbDab entries.

As described below, datasets of structures contained in either CoV-AbDab and/or Thera-SAbDab can be created via the database search interface.

SAbDab contains 370 structures matching Thera-SAbDab entries, four of which are nanobodies and 380 structures matching CoV-AbDab entries, 68 of which are nanobodies.

## UPDATES TO DATA ACCESS

### Search interface

SAbDab can now be searched via a Flask app served by a fast SQL backend to retrieve the full set of structures, specific entries by specifying their Protein Data Bank (PDB) (12) code or to create subsets based on search criteria. Structures can be searched based on the experimental method used to determine the structure, resolution and *R*-factor cutoffs, species of the antibody, type of the antigen (Figure 1), presence of affinity values in the annotation and presence of amino acid residues at specific sequence positions defined using the Chothia numbering scheme.

**Figure 2.**
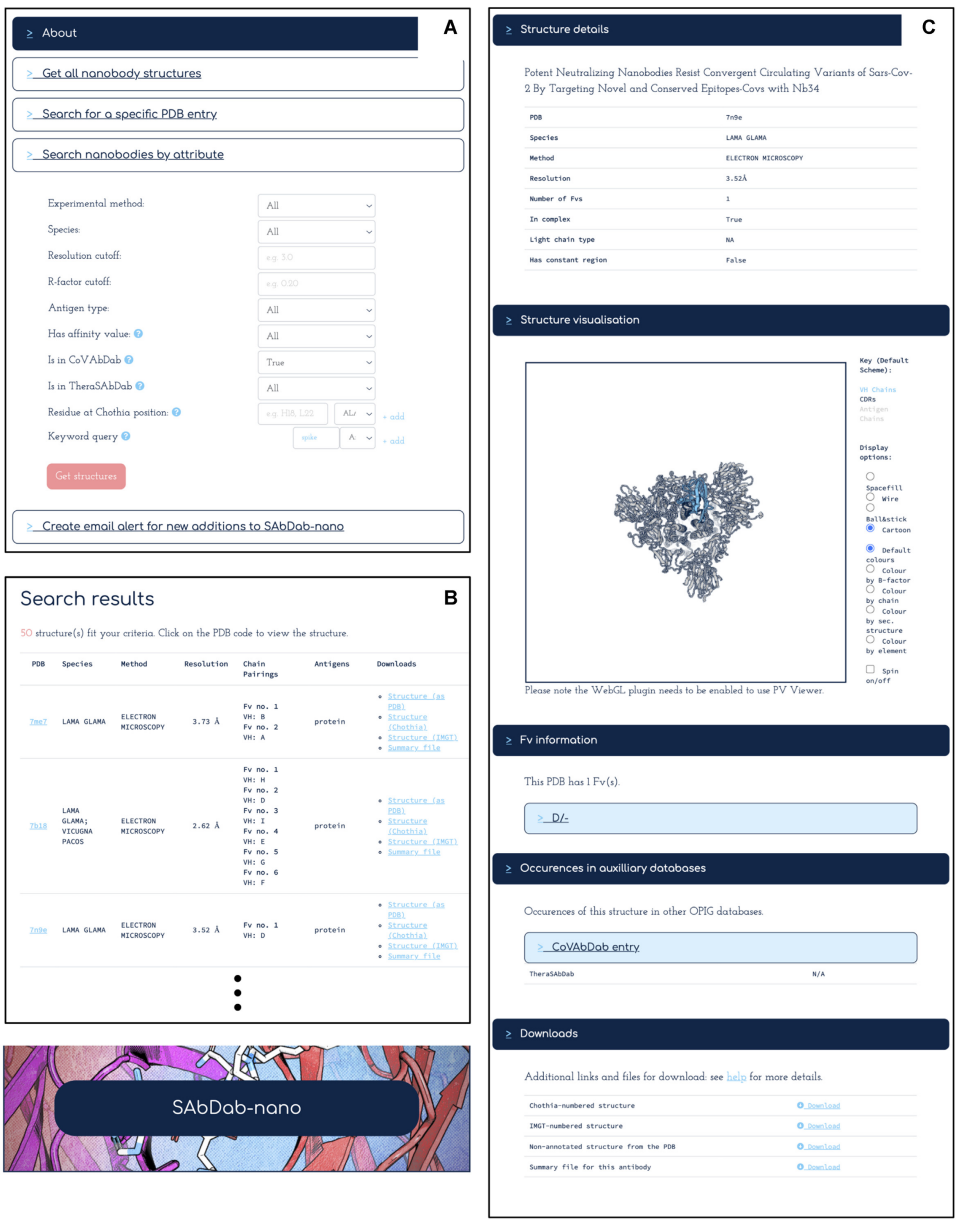
Overview of the SAbDab-nano search interface. (**A**) Example of a search over SAbDab-nano by attributes: The search retrieves all nanobody structures with data in CoV-AbDab, for which the antigen name contains the string ‘spike’. (**B**) The first 3 entries retrieved by the query. (**C**) The structure viewer interface, which shows the annotation on the nanobody structure and download options and provides an interactive 3D visualisation of the structure.

In addition to these search features which were present in SAbDab at the time of its original publication, we now offer the option of searching for nanobody/antibody structures with annotation derived from CoV-AbDab and Thera-SAbDab. Further, a free-text keyword query can be performed over certain annotation fields (antigen, species, publication and structure title), retrieving all structures for which the specified fields contain an exact match to one of up to 10 key strings. Both of these new features improve the ability to create task-specific nanobody and antibody datasets. An example search query is displayed in Figure 2A and B.

Apart from accessing individual structures identified by a search as described below, all structures matching the search query can also be downloaded in bulk as a zipped archive and a summary .csv file containing annotation data.

To help the community stay up-to-date with the release of antibody or nanobody structures of interest, we have now added a feature that generates email alerts based on bespoke user search queries. Using this interface, the search definition is saved on the database server alongside a user-provided email address. After each weekly update, new structures matching the search query are automatically identified and a summary email detailing those structures is sent to the provided address. This feature will, for example, enable users to be immediately notified when a structure of an antibody against an antigen target of interest is added to SAbDab.

### Accessing and downloading individual structures

Individual structures in SAbDab can be accessed *via* the structure viewer interface depicted in Figure 2C, which shows a summary of the structure annotation, an interactive 3D representation of the structure, links to other databases as described above alongside information for each antibody in the structure, including the chain identifiers, CDR sequences and full Fab sequence numbered using the Chothia numbering scheme. Information on the antigen, including antigen sequence, name and chain identifiers, is displayed where the antibody is resolved in complex with an antigen. The user can download the raw structure as deposited in the PDB both with the numbering supplied in the PDB deposited file and with the antibody renumbered according to the Chothia or IMGT numbering scheme (13). The latter numbering scheme is becoming popular in the field of immunoinformatics as it facilitates the comparison of heavy and light chains, as well as different classes of adaptive immune protein (e.g. B-Cell Receptors *vs*. T-Cell Receptors or antibodies *vs*. nanobodies), and was therefore added to SAbDab as an additional download option.

## SAbDab-nano

One of the key updates to SAbDab was the development of SAbDab-nano in order to improve the availability and accessibility of nanobody structures within SAbDab.

From the set of antibodies identified for addition to SAbDab in each weekly update, entries for which at least one antibody has a heavy chain variable domain, but no light chain variable domain are added to SAbDab-nano.

As of September 2021, SAbDab-nano contained 823 structures (see Figure 1), representing 492 nanobodies with non-redundant CDR sequences. This is a more than five-fold increase over the 123 nanobody structures available when SAbDab was initially published, signifying the need for a dedicated sub-database for nanobody structures. SAbDab-nano has grown at an average 3.8 structures/week over the first 36 weeks of 2021. This is only slightly slower than the pace of growth of SAbDab (5.2 structures/week) during the year of its release (2013), further showing the growing need for a more dedicated resource.

The majority of SAbDab-nano entries are resolved in complex with a protein antigen, 78% of entries. This is a far larger proportion than for SAbDab, where only 56% entries contain a protein antigen (see Figure 1). This difference in the distribution of antigen types between SAbDab-nano and SAbDab is primarily due to a shift over last decade in the types of antibody structures which are experimentally determined; whereas in 2013, protein antigens were present in 55% of PDB-deposited antibody structures, in 2020 this ratio had risen to 75%. As >50% of nanobody structures have been released since 2018, SAbDab-nano reflects this trend in its overall composition.

### Comparison of SAbDab-nano to other nanobody resources

There are other databases and resources compiling nanobody data. However, to our knowledge no other resource provides nanobody structures in a continuously updated and comprehensively annotated format.

There are several databases compiling nanobody sequences (but not structures) from a variety of data sources. INDI (14) contains more than 11 million nanobody sequences, including, at time of writing, sequences derived from 805 PDB structures, but does not provide experimentally resolved structures. sdAb-DB (15) contains 1452 single-domain antibody sequences, of which 195 are derived from experimentally resolved structures, but similarly does not provide the corresponding structures.

There are two currently accessible resources containing annotated nanobody structures. Zavrtanik and Hadži (16) compiled a static, non-redundant set of 123 nanobody structures in complex with antigens, derived from 217 PDB deposited structures. Abybank-AbDb (17) contains a set of 347 nanobody structures, which can be downloaded in bulk, but over which no additional search can be performed.

Further, nanobody structures are of course present in the PDB (12) and the IMGT/3Dstructure-DB (18), but are not annotated as such and thus cannot be retrieved trivially from these databases.

## CONCLUSION

SAbDab continues to be updated weekly and represents the most thoroughly annotated antibody structure database from which researchers can quickly create custom datasets for their studies. Searching SAbDab is now more powerful and faster, with new connections to auxiliary databases that catalogue therapeutic and antigen-specific antibodies. These links will continue to be extended as more such databases become available. By creating SAbDab-nano, an explicit nanobody-tracking sub-database, we have provided an additional resource for researchers investigating the structural properties of this emerging class of bio-therapeutics.

SAbDab and SAbDab-nano can be accessed freely online under a CC-BY 4.0 license at opig.stats.ox.ac.uk/webapps/newsabdab/ or at opig.stats.ox.ac.uk/webapps/newsabdab/nano respectively. Both are available under an academic or commercial license as part of the SAbBox virtual machine at http://opig.stats.ox.ac.uk/sabbox, which gives access to the SAbDab Application Programming Interface (API) for more powerful dataset creation.
